# 6Pgdh polymorphism in wild bulb mite populations: prevalence, environmental correlates and life history trade-offs

**DOI:** 10.1007/s10493-024-00909-4

**Published:** 2024-04-10

**Authors:** Pranav Unnikrishnan, Szymon Grzesik, Magdalena Trojańska, Beata Klimek, Agata Plesnar-Bielak

**Affiliations:** 1 Faculty of Biology, Institute of Environmental Sciences, Jagiellonian University, ul. Gronostajowa 7, 30-387 Kraków, Poland; 2 Department of Pathobiology, Institute of Microbiology, University of Veterinary Medicine, 1210 Vienna, Austria

**Keywords:** *Rhizoglyphus robini*, 6-Phosphogluconate dehydrogenase, Genetic polymorphism, Balancing selection, Metabolic gene, Genotype–environment interaction for fitness

## Abstract

Genetic polymorphism in key metabolic genes plays a pivotal role in shaping phenotypes and adapting to varying environments. Polymorphism in the metabolic gene *6-phosphogluconate dehydrogenase* (*6Pgdh*) in bulb mites, *Rhizoglyphus robini* is characterized by two alleles, S and F, that differ by a single amino acid substitution and correlate with male reproductive fitness. The S-bearing males demonstrate a reproductive advantage. Although the S allele rapidly fixes in laboratory settings, the persistence of polymorphic populations in the wild is noteworthy. This study examines the prevalence and stability of *6Pgdh* polymorphism in natural populations across Poland, investigating potential environmental influences and seasonal variations. We found widespread *6Pgdh* polymorphism in natural populations, with allele frequencies varying across locations and sampling dates but without clear geographical or seasonal clines. This widespread polymorphism and spatio-temporal variability may be attributed to population demography and gene flow between local populations. We found some correlation between soil properties, particularly cation content (Na, K, Ca, and Mg) and *6Pgdh* allele frequencies, showcasing the connection between mite physiology and soil characteristics and highlighting the presence of environment-dependent balancing selection. We conducted experimental fitness assays to determine whether the allele providing the advantage in male–male competition has antagonistic effects on life-history traits and if these effects are temperature-dependent. We found that temperature does not differentially influence development time or juvenile survival in different *6Pgdh* genotypes. This study reveals the relationship between genetic variation, environmental factors, and reproductive fitness in natural bulb mite populations, shedding light on the dynamic mechanisms governing *6Pgdh* polymorphism.

## Introduction

Balancing selection is the process through which polymorphism within a population is actively maintained over generations. Such maintenance of genetic variation is particularly important in genes that govern key metabolic traits, as variation in these genes has the potential to significantly impact the organism’s fitness and chances of survival (Koshiba et al. 2020; Whitt et al. 2002). Despite the general conservation of metabolic genes (Mukherjee et al. 2018; Kapahi et al. 2010), the presence of functional differences and selective pressures acting on them suggest, in some cases, the action of balancing selection. Metabolic genes can affect multiple aspects of an organism’s physiology and many traits. Therefore, balancing selection on these genes is likely to take the form of antagonistic pleiotropy, where a gene affects two or more traits of an organism with opposite effects on fitness (Stearns 1998). In genes with multiple functional alleles under antagonistic pleiotropy, one of the alleles is beneficial for one trait while the other for another trait (oftentimes with additional detrimental effects on fitness), thus creating a tradeoff between different fitness components (Meyer and Zanger 1997; Kozyra et al. 2017; Di Bartolomeo et al. 2020). Antagonistic pleiotropy is already a recognized mechanism for the maintenance of polymorphism (Hedrick 1999).

This mechanism becomes even more intriguing when we consider the complex interplay of environmental factors (Brown and Kelly 2018; Mérot et al. 2020) where antagonistic pleiotropy might be present in some environments, but not in others. Different enzyme variants can be favored at different environments, resulting in selection on such enzyme variants and influencing the geographic patterns of genetic diversity. For example, in *Drosophila serrata* (Rusuwa et al. 2022), the polymorphism in a single gene influencing circular hydrocarbon profile (CHC) is maintained in warm and humid climates but not in hot and dry ones. This is because one of the alleles is associated with male reproductive advantage, but lowers female desiccation resistance, resulting a trade-off between male reproduction and female stress response (termed also sexually antagonistic pleiotropy) and driving balancing selection in specific climate conditions (Rusuwa et al. 2022). The influence of environmental heterogeneity on functional polymorphism in metabolic genes was shown by Kerwin et al (2015) on the gene involved in glucosinolate production in *Arabidopsis thaliana*, which showed that no allele consistently outperformed another in different environments, emphasizing the importance of environmental heterogeneity as an evolutionary force. Understanding the relationship between environmental factors and the functioning of enzyme variants is essential for gaining insight on how organisms adapt to their environment, utilize resources (Vieille and Zeikus 2001), defend against diseases (Hollman et al. 2016), and evolve (Rix et al. 2020).

This study focuses on the metabolic gene *6-Phosphogluconate dehydrogenase* (*6Pgdh*), which is also a target of sexual selection in bulb mite, *Rhizoglyphus robini*. 6Pgdh is one of the key enzymes in the Pentose Phosphate Pathway (PPP), an alternative to glycolysis. It produces NADPH and ribose-5-phosphate used in the synthesis of fatty acids, sterols and nucleotides (Ge et al. 2020). PPP is also a source of amino acid and vitamin B6 precursors (Tambasco-Studart et al. 2005). The pathway is essential for energy metabolism of a cell and plays an important role in stress response to abiotic factors such as temperature, salinity, drought, etc. (Fahrendorf et al. 1995; Krüger et al. 2011; Hou et al. 2007). The expression levels and activities of PPP enzymes are associated with environmental factors (Watts and Lawrence 1990). Environmental heterogeneity has been shown to drive patterns of polymorphism in the *6Pgdh* and some other genes involved in PPP in organisms ranging from invertebrates (González-Ruiz et al. 2023) to plants (Landi et al. 2021). However, to really understand how selection of genes involved in PPP is driven by environment, we need to connect geographical and temporal patterns of polymorphism with phenotypic differences between genotypes and environmental background of these differences.

*6Pgdh* coding sequence in bulb mites consists of 486 amino acids and is divided by four introns. While four SNPs have been found in the coding sequence (Skwierzyńska and Plesnar-Bielak 2018), only one is associated with amino acid substitution (arginine to methionine). The two alleles of *6Pgdh*, S and F correlate with reproductive fitness of males. Males bearing the S allele gain higher reproductive success compared to males with the F allele (Konior et al. 2006; Łukasik et al. 2010), due to higher sperm production and copulation frequency (Skwierzyńska and Plesnar-Bielak 2018). Female fitness seems to be independent of the *6Pgdh* genotype, but the S-bearing males reduce the fitness of their female partners, even though the exact mechanism behind it is unknown (Konior et al. 2006). As expected from the advantage in male competition, the S allele rapidly fixes in laboratory conditions, but surprisingly, the polymorphism seems to be maintained in some natural populations (Konior et al. 2006; Łukasik et al. 2010, personal observations). There is very limited knowledge regarding the ecological conditions linked to this polymorphism and we hypothesize the active maintenance of polymorphism of this metabolic gene is environment-dependent. Nevertheless, a systematic study on the patterns of *6Pgdh* variation in natural bulb mite populations has not been conducted before so it is not known how common and stable over time the polymorphism is.

Here, we study several natural bulb mite populations to look into the amount of polymorphism and its geographical variation across Poland. To start with, we aim to correlate *6Pgdh* polymorphism levels with latitude and longitude, most important macro-climatic factors, as well as with local soil properties. Moreover, we investigate seasonal variation in *6Pgdh* frequencies, by investigating frequency shifts between spring and autumn. Finally, we explore fitness differences between *6Pgdh* genotypes at different temperatures. We test if the allele providing advantage in male-male competition has antagonistic effects on life history traits and if these effects are temperature-dependent, as earlier studies have suggested the effects of temperature on PPP (Kauffman et al. 1969) and on *6Pgdh* allele frequencies (Plesnar-Bielak et al. 2020). So, we further complement our field study with experimental fitness assays at different temperatures. The expression of the S allele, that conveys reproductive advantage to males, might be associated with energetic costs that could result in reduced juvenile survival and/or longer development time for individuals bearing this allele.

## Methods

### Study species

The bulb mites, *Rhizoglyphus robini* (Acari: Acaridae) are common pests with cosmopolitan geographical distribution. They inhabit subterrain parts of *Lilliaceae* and other plants (Díaz et al. 2000). The life cycle of *Rhizoglyphus robini* consists of egg, larva, protonymph, tritonymph, and adult stages, with a facultative migratory stage of deutonymph, which develop from protonymphs when conditions are unfavorable (overcrowding, low food availability etc.). The bulb mites do not enter a diapause (Gerson et al. 1991), remaining active throughout the year. However, their activity might be reduced during colder months.

*Rhizoglyphus robini* is characterized by high promiscuity (Radwan and Siva-Jothy 1996) and is used as model species in sexual selection studies (e.g. Smallegange and Coulson 2011; Jarzebowska and Radwan 2010; Plesnar-Bielak et al. 2012; Łukasiewicz et al. 2017; Parrett et al. 2022). Both males and females mate multiply, with mating frequency depending on environmental conditions (Gerson and Thorens 1982). While male fitness increases with the number of copulations, multiple mating is associated with fitness cost to females, signifying sexual conflict (Tilszer et al. 2006). The amount of male harm has been shown to depend on a male’s *6Pgdh* genotype, such that mating with males bearing the S allele is associated with higher cost than mating with a male lacking this allele (Konior et al. 2006). This effect might, at least to some extent, be caused by higher ability of the S-bearers to exert more frequent copulations of females (Skwierzyńska and Plesnar-Bielak 2018), but the actual physiological mechanism of female fitness reduction is not clear.

### DNA extraction and *6Pgdh* genotyping

DNA was extracted from individual mites. Each individual was placed in 1% chelex solution (40 μl) and was crushed. Then 3 μl of proteinase-K (EurX) was added, and the mixture was incubated in a thermocycler (10 min 94 °C, 15 min 75 °C).

The *6Pgdh* genotyping was done using Real-Time PCR with fluorogenic TaqMan probes (Thermofisher Scientific) specific for the missense single nucleotide polymorphism determining the F and S alleles. The Bio-Rad CFX96 Real-Time PCR detection system was used for the genotyping. A TaqMan Genotyping Master Mix (Thermofisher Scientific) and Custom Genotyping Assay that included allele-specific primers and fluorescent probes were mixed in 10:1 ratio. 5.5 μl of such a mix and 4.5 μl of DNA were put in a 96 well plate for genotyping. PCR was performed in 41 cycles (15 s 95 °C, 1 min 60 °C).

### The patterns of *6Pgdh* polymorphism in the wild

Population sampling was carried out in Poland, which has a clear gradient of climatic and environmental conditions such as temperature, precipitation, air pressure, etc. from south-west to north-east (Błaś and Ojrzyńska 2024; Blazejczyk 2006) that might affect *6Pgdh* frequencies. Moreover, there is some record of variation in the level of *6Pgdh* polymorphism in Poland and substantial genetic diversity within populations with little structuring between populations in this region (Kolasa et al. unpublished; Boroń et al. unpublished; Przesmycka and Radwan 2023). Sampling was done between October 2021 and December 2022. The main sampling was done in late Spring/Summer (May, June) with some locations sampled also in Autumn (October, November) to see how stable *6Pgdh* frequencies are across seasons.

Samples of bulbs of different plant species were collected from private gardens and botanical gardens across different regions in Poland (Table 1) and checked for the presence of mites. Between 2 and 6 plant bulbs together with soil samples (taken only during Spring sampling and for a subset of samples) were collected per location, depending on availability.

**Table 1:** Locations (with latitude and longitude) from which the samples were collected across Poland and the respective seasons during sample collection

Location	Latitude	Longitude	Season	Number of mites genotyped	F frequency	Heterozygosity	H–W test, P-value
Stanislaw Gorny	49.91062	19.62935	Autumn	38	0.145	0.184	2.005 0.157
Krakow OB	50.06368	19.95549	Autumn	39	0.051	0.103	0.216 0.642
Mikolow	50.18060	18.82853	Autumn	52	0.596	0.538	0.734 0.392
Rudawa	50.12200	19.71217	Autumn	35	0.200	0.229	2.501 0.114
Lublin	51.29331	22.53616	Autumn	45	0.145	0.178	0.782 0.377
Marszyce	50.18099	19.85584	Autumn	54	0.130	0.148	4.733 0.029
Warszawa	52.25531	21.02248	Autumn	42	0.071	0.048	8.494 0.004
Brzeg	50.87413	17.46600	Spring	9	0.333	0.444	3.55e−15 1
Glucholazy.1	50.33271	17.37789	Spring	32	0.562	0.375	1.824 0.177
Brody	49.88037	19.72432	Spring	28	0.250	0.286	1.479 0.224
Rudawa	50.12200	19.71217	Spring	35	0.242	0.314	0.701 0.402
Piekary Slaskie	50.34695	18.98586	Spring	25	0.320	0.400	0.161 0.688
Stanislaw Gorny	49.91062	19.62935	Spring	33	0.576	0.424	0.571 0.449
Krakow	50.06368	19.95549	Spring	36	0.194	0.056	21.026 4.53e−06
Lublin.1	51.29331	22.53616	Spring	30	0.033	0.067	0.069 0.793
Lublin.2	51.29331	22.53616	Spring	6	0.416	0.500	0.005 0.944
Bory Tucholskie	53.61859	18.16236	Spring	27	0.000	0.000	n.a. 1
Poznan	52.41385	16.92981	Spring	28	0.054	0.107	0.170 0.680
Warszawa	52.25531	21.02248	Spring	28	0.232	0.179	6.226 0.013
Glucholazy.2	50.33271	17.37789	Spring	30	0.500	0.000	41.589 1.12e−10
Przysieki	49.74042	21.38654	Spring	32	0.000	0.000	n.a.
Mikolow	50.18060	18.82853	Spring	38	0.026	0.000	9.248 0.002
Kepa Slupska	54.41783	17.05663	Spring	29	0.138	0.138	3.785 0.052
Lublin.3	51.29331	22.53616	Spring	24	0.250	0.250	2.454 0.117

The F-allele frequency, heterozygosity measures and results from the Hardy Weinberg test for each location are also shown

In the lab, bulb mites, if present, were transferred to plastic containers (diameter ≈ 2.5 cm) with plaster of Paris soaked with water (which are standard containers to keep large groups of mites). They were kept at 12 °C and fed powdered yeast ad libitum. Ca. 40 individuals from each sample (location, see Table 1) were genotyped within 2 months after collection to ensure that the individuals collected as juveniles reached adulthood.

### Climate data

Climate data was obtained from the KNMI climate explorer website (https://climexp.knmi.nl). Daily values of mean surface temperature (in °C) and precipitation/rainfall (in mm/day) were obtained from the E-OBS database, with 0.25° regular grids. Daily climatic values were obtained for each location using their coordinates (from the grid they belonged to) for 60 days before the bulb collection date. The values were then averaged for each location (so that we obtained a mean for a 60-day long period before collection) and used for the analysis. Using the same procedure, we also calculated average surface temperatures and rainfall values for the 30 and 90-day periods before collection.

### Soil analyses

Soil samples were collected from sampling points (10 sampling points) near the plant using teaspoons and ensuring that the points were within 1 m^2^ of the plant and were kept in 12 °C for analysis. The dry weight (DW) of the soil samples was determined by measuring mass loss (water) after soil samples dried at 105 ± 1 °C for 24 h. Next, the organic matter content (OM) in soil dry weight was determined as the mass loss on ignition at 550 ± 1 °C for 24 h. The water holding capacity (WHC), which is the amount of water that a given soil can hold without leaking, was measured by a standard gravimetric method after soil soaking for 24 h in net-ended plastic pipes immersed in water. The organic carbon (C), total nitrogen (N), and total sulfur (S) were analyzed by dry combustion of ca 10 mg milled soil samples with an elemental analyser (Vario El III, Elementar Analysensysteme GmbH). The soil pH was measured in air-dried subsamples (2 g) shaken in deionised water (1:10 w:v) for 1 h at 200 rpm (pH-meter with glass electrode).

The total element concentrations, that is phosphorus (P), calcium (Ca), potassium (K), magnesium (Mg), manganese (Mn), and sodium (Na) in each soil sample were determined after wet digestion of ca 0.5 g of DW in 10 ml of SupraPure-concentrated HNO_3_ and HClO_4_ (7:1 v/v) (Sigma-Aldrich). A flow injection analyser (FIA compact, MLE, Radebeul, Germany) was used to determine the P content. The total concentrations of the other elements were measured using atomic absorption spectrometry (AAS) with a flame nebulizer (Perkin-Elmer, AAnalyst200, Waltham, Massachusetts, USA). The accuracy of the mineralization process was determined using blank samples as well as standard certified material (CRM025-050, Sandy Loam 8, RT Corp.). Each analysis was performed in two subsamples from each soil sample, and the data were averaged and expressed based on the dry weight of the soil.

### Laboratory population

For the life-history fitness experiments, we used a population enriched in the F allele that was established from a field population obtained in July 2020 from Łazany (49.9476, 20.1535) near Kraków. Several dozens of individuals collected from an onion were placed in a common container with powder yeast that served as food. Such obtained population was kept at 8 °C, with the exception of a 1-week period after we finished collecting individuals, when it was moved to 24 °C to let juvenile individuals develop so that population would expand. The F-increased population was created in spring 2021, when the F allele frequency in the source population was about 0.23. To do it, we randomly paired virgin females and males from a source population. After the pairs mated and females laid eggs, both parents were genotyped. Eight offspring from pairs with parents having at least 2 copies of F allele (either both parents FS, or one FF and one SS, or one FF and one SF, or both parents FF) were transferred as larvae/protonymphs to a common container to establish a population with increased F allele frequency. We used two containers (with offspring from the same parental pairs moved to both of them) that established two subpopulations that were mixed and divided again after ca. 2 months. The population was let to expand freely for ca. 2 months at 24 °C (which corresponds to 3–4 mite generations), before it was moved to 12 °C to elongate generation time, slowing down population’s evolution and the loss of the F allele. At all these stages the population was kept at > 90% humidity and constant darkness, with powdered yeast provided ad libitum as a food source.

### Development time

**Fig. 1:**
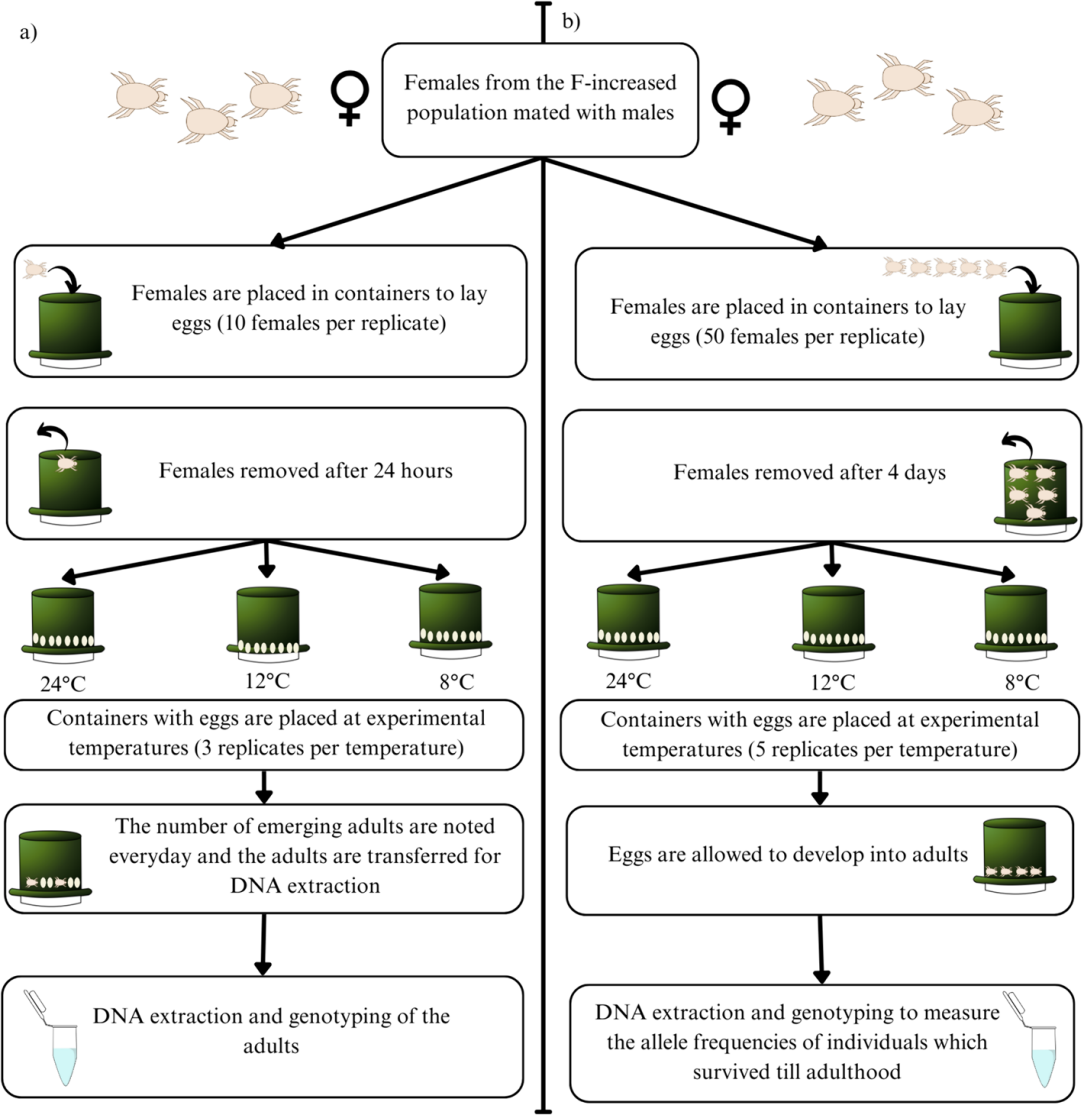
Methods of laboratory fitness assays—**a** Development time, **b** Juvenile survival

Development time of the individuals with different genotypes was measured at three temperatures, 24 °C (standard temperature in which the laboratory populations are reared), 12 °C (average yearly ground temperature at 5–10 cm depth in Poland) and 8 °C (low temperature relevant to colder months of sampling) with three replicates per temperature (see Fig. 1). Per each replicate, ten females were randomly selected from the F-increased population were kept in containers for 24 h to lay eggs. After the females were removed, the containers with the eggs were placed to experimental temperatures. The eggs were allowed to develop. When they reached the stage of tritonymph (last juvenile stage), they were checked every 24 h for emerging adults. Adults that emerged were taken out from the containers and date of emergence and sex were noted. Then, the individuals were genotyped for *6Pgdh*. The checks continued until all the adults emerged.

### Juvenile survival differences between genotypes

Juvenile survival differences were also tested at 24 °C, 12 °C and 8 °C. For the assay, 50 females were put in a common container (five replicates per temperature) and allowed to lay eggs at 24 °C for 4 days, after which they were removed (see Fig. 1). The containers were then transferred to their respective experimental temperatures (24 °C, 12 °C and 8 °C). After all the adults emerged, around 40 individuals from each replicate were genotyped for the *6Pgdh*. We calculated the frequencies of the F allele at each temperature and used them as a proxy of juvenile survival differences between genotypes. If juvenile survival of individuals with different alleles is temperature-independent, we expect that allele frequencies in adults do not differ between temperatures. A higher frequency of a certain allele at a given temperature, indicates higher survival of the individuals bearing this allele.

### Statistical analysis

*6Pgdh* genotype frequencies in field samples were tested for Hardy–Weinberg equilibrium with likelihood ratio test implemented in Hardy–Weinberg package in R (Graffelman and Weir 2016). The frequencies from the samples collected in spring (when most of the samples were collected) were checked for their relationship with latitude and longitude of the location to look for geographical cline *6Pgdh* polymorphism. We applied a quasibinomial model accounting for overdispersion (using glm function in R v3.6.1) with a vector of S and F allele counts at each location as a response variable and latitude and longitude as independent variables. For plotting the data points on the map of Poland, QGIS (v3.34.0-Prizren) was used along with the map shape file obtained from GADM data (v4.1).

Both mean temperature and precipitation levels were highly correlated (r_22_ = 0.57, p < 0.01 for a 60-day-long period). They were analyzed in separate quasibinomial models with a vector of S and F allele counts from each location as the response variable and the climatic variable (mean surface temperate or precipitation) as the predictor variable. The models were rerun for data averaged for 30 and 90 days. Since the results were qualitatively the same, we present only the analyses for 60 days.

To test for a correlation between 6Pgdh frequencies and soil characteristics, we first summarized soil parameters with Principal Component Analysis. Then, we ran a generalized linear model with a vector of S and F allele counts at each location as a response variable and PC1 and PC2 as independent variables. Again, quasibinomial distribution was used to account for overdispersion in our data.

To check how genotype affects development time at different temperatures, we used a linear mixed model fit with the number of days taken for development (transformed with square root) as the response variable and with genotype and temperature (factor) as the dependent variables and population ID as random factor. We also checked to see if the effect of sex of the individuals was important to the model using AIC scores, but the effect of sex did not improve the model and the conclusions remained unchanged and hence the effect of sex was removed from the main model. The function lmer was used for the analysis in R (the package lmertest, lme4, v1.1-26).

The allele frequencies of the individuals that survived to adulthood at each temperature were obtained from the juvenile survival experiment. To analyze the data, a binomial model was used with a vector of S and F allele counts in each replicate as the response variable and temperature as the dependent variable using the glm function in R (glm2 package, v1.2.1).

## Results

### The patterns of *6Pgdh* polymorphism in the wild

**Fig. 2:**
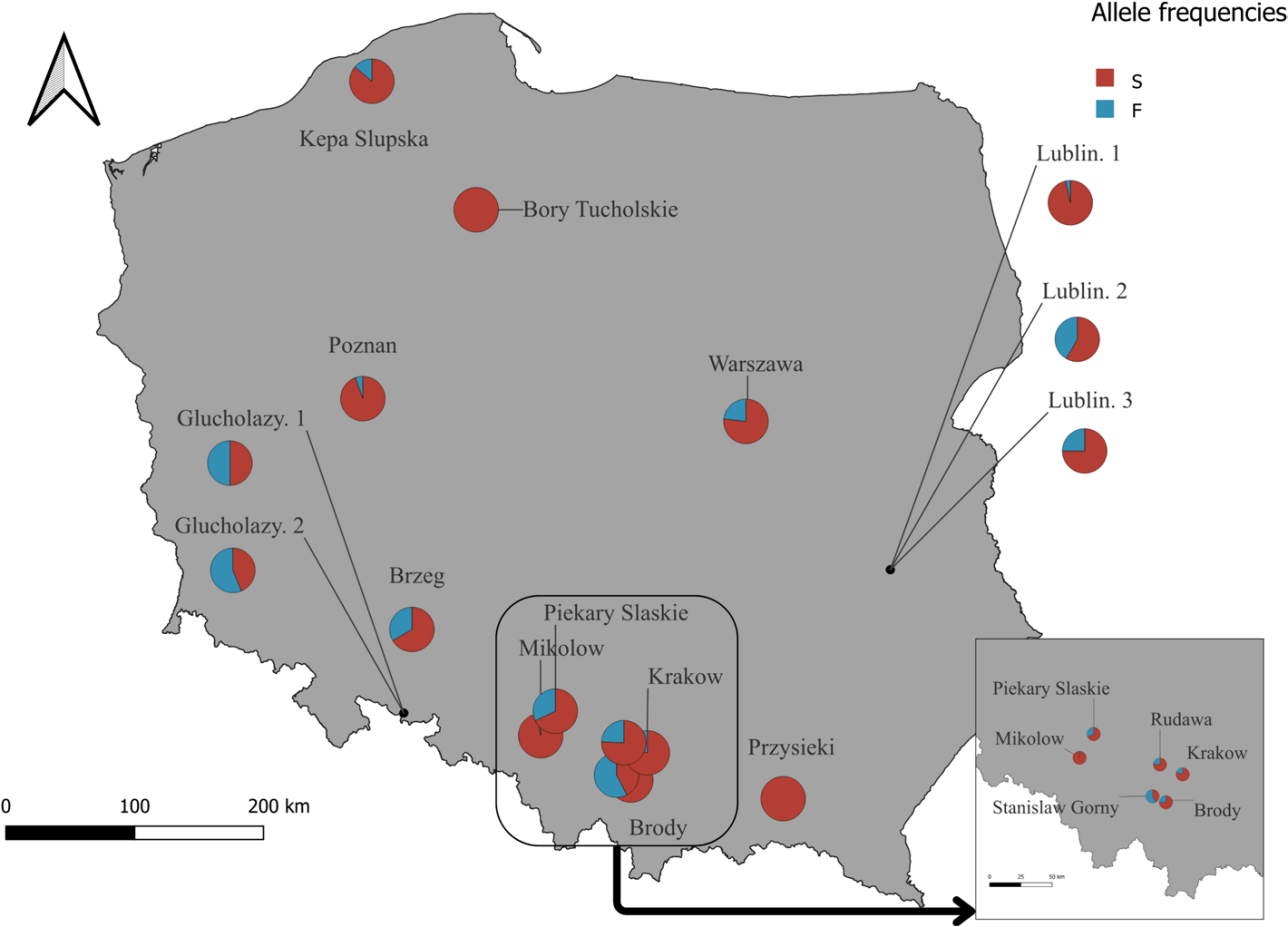
F-allele frequency of natural bulb mite populations at different locations in Poland in spring

**Fig. 3:**
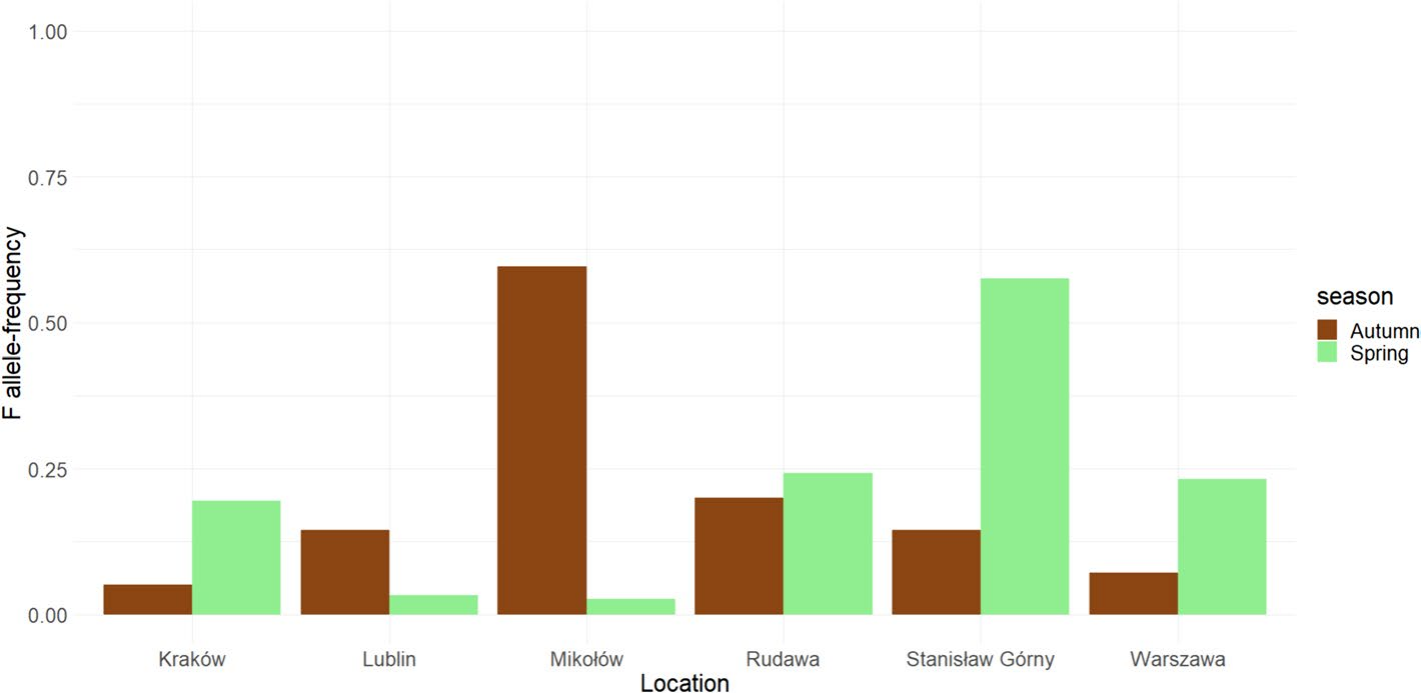
F-allele frequencies between spring and autumn at various locations in Poland

We found *6Pgdh* polymorphism in a majority (15 out of 17) of populations (Fig. 2), with only two of them having just one allele (S). In polymorphic populations, F-allele frequencies varied from 0.026 to 0.60, with a mean frequency of 0.23 (SD = 0.19). Mean frequency was similar for the samples collected in Autumn (mean ± SD 0.191 ± 0.18) and Spring (0.243 ± 0.19). *6Pgdh* allele frequencies varied a lot between Autumn and Spring, but the changes were not consistent in their direction, with F frequency increasing in Spring in some locations, but decreasing in others (Fig. 3). Similarly, samples taken from the same botanical garden substantially differed in allele frequencies, even in the same season. Eighteen samples were in Hardy–Weinberg equilibrium. In 6 samples (Table 1), we found deviation from Hardy–Weinberg equilibrium and observed heterozygosity was lower than expected in all these cases. We did not find evidence for geographical clines in *6Pgdh* frequencies in Poland (longitude: effect estimate − 0.285, t = − 1.78, p = 0.089, latitude: effect estimate − 0.406, t = − 1.88, p = 0.075). Similarly, we did not find any influence of climatic variables in 6Pgdh allele frequencies (temperature: effect estimate 0.132, t = − 1.27, p = 0.219, precipitation: effect estimate − 0.402, t = − 0.62, p = 0.543). The data from the 90 days and 30 days interval provided with similar results (not shown).

### The association of *6Pgdh* polymorphism with soil properties

**Fig. 4:**
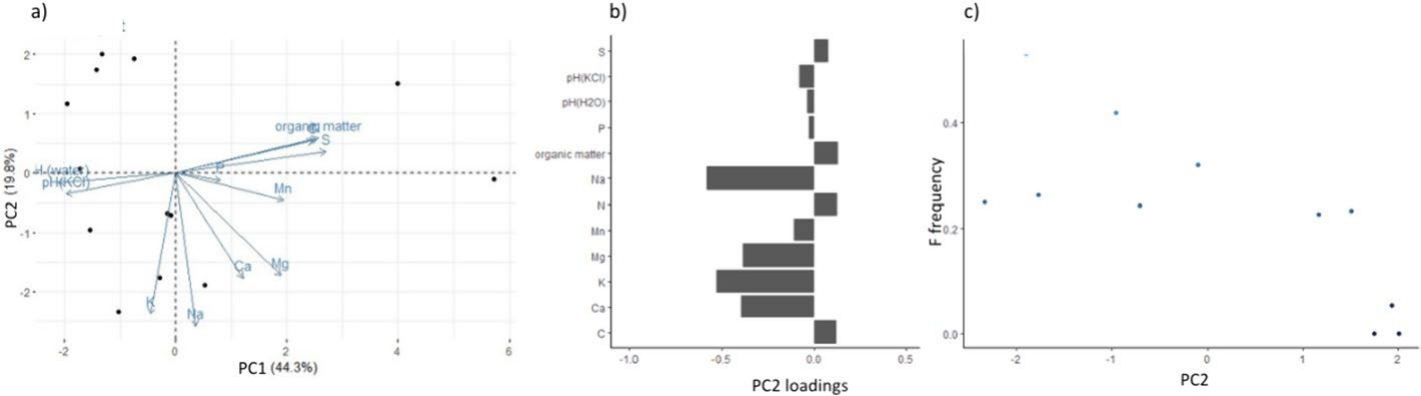
**a** The results from the PCA showing the PC1 and PC2 axes explaining 44.3% and 19.8% of the variance in soil patterns respectively. **b** The inverse relationship of the second PC axis with the Na, Mg, K and Ca content of the soil. Units: S (%), P (%), organic matter (%), Na (mg kg^−1^), N (%), Mn, Mg, K, Ca (mg kg^−1^), C (%). All data are expressed per dry soil mass. **c** Negative relationship between PC2 and F-allele frequency

PC1 and PC2 explained 44.3 and 19.8% of the total variance in soil parameters, respectively (Fig. 4a). PC1 was mainly influenced by organic matter, S, N and C content (Fig. 4b). Higher PC1 values were also associated with lower pH. PC2 values were negatively correlated with Na, K, Ca and Mg content. We found a significant negative relationship between F allele frequencies and PC2 (t_11_ = − 2.99, p = 0.017), but not PC1 (t_11_ = 1.584, p = 0.152). (Fig. 4c).

### Laboratory fitness experiments

**Fig. 5:**
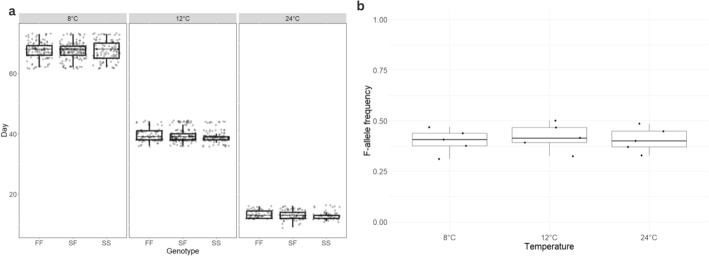
**a** Development time for bulb mites with different genotypes of the *6Pgdh* allele at different temperatures. The boxplot shows the median along with the interquartile range. The points represent the spread of individual data points. **b** F-allele frequencies of the juveniles that survived at each temperature. The boxplot shows the median along with the interquartile range. The data points represent the F-frequency of the replicates

Development time increased with decreasing temperature (F_2; 8_ = 7521.85, p < 0.01). There was no difference in development time between genotypes (F_2; 953_ = 1.53, p = 0.22) or genotype by temperature interaction (F_4; 954_ = 1.33, p = 0.25) (Fig. 5). The effect of sex was also not significant (F_1; 950_ = 1.31, p = 0.22).

F allele frequencies did not differ between groups developed at different temperatures (effect estimate 0.002, z = 0.17, p = 0.86), indicating there is no temperature-dependent difference in juvenile survival between genotypes (Fig. 5).

## Discussion

Genetic polymorphisms in key metabolic genes can potentially influence a wide range of phenotypic traits and may therefore be important for adaptive polymorphism. *6Pgdh* is an example of such a gene with different allelic variants influencing fitness in vertebrates (Rivera et al. 2006; Chen et al. 2023), plants (Oostermeijer et al. 1995) and invertebrates (Begun and Aquardo 1994; Kilias and Alahiotis 1985), including bulb mites (*Rhizoglyphus robini*). The maintenance of such polymorphism under natural conditions is surprising, particularly in the case of bulb mites, given the strong reproductive advantage of the S allele leading to its rapid fixation in the laboratory. In some environments, however, reproductive advantage may be balanced by metabolic costs that could lead to a trade-off between sexual and non-sexual fitness, leading to the maintenance of stable polymorphism under these conditions (Robinson et al. 2006; Höglund et al. 1998). Our study investigates the abundance of *6Pgdh* polymorphism in natural populations, explores potential environmental factors influencing these patterns in the wild, and examines potential trade-offs in laboratory settings.

We show that *6Pgdh* polymorphism is indeed common in natural populations. The majority of screened populations were *6Pgdh*-polymorphic, but the actual allele frequencies varied. The abundance of polymorphism aligns with certain observations reported in prior studies (Łukasik et al. 2010). For example, a natural population from Poland found by Konior et al. (2006) was polymorphic in respect to *6Pgdh* with F-allele frequency of 0.11. Screening of ten populations in Poland by Skwierzyńska and Plesnar-Bielak (2018) found only one polymorphic population, suggesting *6Pgdh* polymorphism to be rare. However, the search was aimed at finding a population of a relatively high polymorphism level to use in laboratory experiments. Hence, the sampling might have not been suitable for detecting moderate F frequencies, which were not uncommon in the current study.

*6Pgdh* frequencies varied between populations and sampling dates, but there was no seasonal or latitudinal pattern. Neither surface temperature nor precipitation/rainfall were able to explain the variation in *6Pgdh* allele frequencies. The high variability of *6Pgdh* allele frequencies is supported by previous observations in bulb mites. For example, the F frequency in a natural population was 0.34 in 2003, but it decreased below 0.05 at the same site a year later (Łukasik et al. 2010). The lack of geographic, macroclimatic or seasonal patterns suggests that perhaps factors other than temperature and rainfall may play a more significant role in shaping the frequencies of *6Pgdh* alleles or that the effect of these variables may occur at a much finer scale. These fine scale effects might indeed be important; for example, plant cover may significantly affect shading and hence drastically affect both temperature and humidity (Procházka et al. 2011; Zhang et al. 2013). Similarly, since our sites were located in gardens, plant watering was likely to overwrite the effects of large-scale precipitation patterns. On the other hand, our results from the laboratory experiments do not support temperature’s role in shaping *6Pgdh* frequencies in the bulb mite. We found no evidence that temperature differentially affects two life-history traits: development time and juvenile survival, in individuals with different *6Pgdh* genotypes. This suggests that temperature does not have an effect on the allele fitness or the distribution of alleles on neither microhabitat level (life-history assays) nor macrohabitat scale (field study). Similarly, a prior laboratory investigation demonstrated that there is no reversal in F-allele fitness across temperatures, supporting our conclusion (Plesnar-Bielak et al. 2020). While the results from the laboratory studies suggest that temperature doesn’t contribute to the levels of *6Pgdh* allele frequencies, we cannot rule out other factors as demonstrated in other species. For example, two studies in *Drosophila* (*D. melanogaster* and *D. simulans*) showed clear latitudinal clines of *6Pgdh* allele frequencies in different regions across the world, likely associated with climatic conditions (Oakeshott et al. 1983; Begun and Aquadro 1994). Similarly, latitudinal clines in *6Pgdh* frequencies have been associated with other selective factors such as water salinity in Atlantic killifish (*Fundulus heteroclitus*) (Powers et al. 1986). Stockwell and Mulvey (1998) also explicitly demonstrated that it was water salinity, and not temperature, that influenced the polymorphism levels in white sands pupfish, *Cyprinodon tularosa* (Stockwell and Mulvey 1998).

Our study found a significant effect of soil properties on the level of polymorphism, with higher amounts of cations (Na, K, Ca and Mg) in the soil corresponding to higher frequencies of the F allele. Bulb mites, being subterranean organisms reliant on soil, can experience direct or indirect effects of soil composition on their physiology. Soil properties can affect plant diversity and soil fauna indirectly (Kudureti et al. 2023). Indeed, evidence for soil properties differentially affecting fitness of multiple mite species have already been observed. Soil properties such as pH, nitrogen and carbon content, among other variables have been shown to affect community composition (Nielsen et al. 2010) and diversity (de Moraes et al. 2011) in oribatid mite group, suggesting they drive fitness differences at inter-species level. Our results suggest that soil properties can differentially affect mite fitness at the intra-species level too. The effects of soil could be mediated by factors like vegetation or soil microbial community composition (Li et al. 2023; Pineda et al. 2017). For example, host-microbiome interactions in bulb mites affect nutrition (Zindel et al. 2013), which in turn impacts fitness and traits like development rate and body size (Leigh and Smallegange 2014). Nutritional conditions can also influence fitness of different alleles of the same metabolic genes. It has been shown in *Drosophila melanogaster*, where diet quality affected fitness of the allelic variants of the “foraging” gene (Burns et al. 2012). In general, the patterns of *6Pgdh* polymorphism could be associated with environmental quality, mediated by the relationship between soil properties and microbiome. However, resolving this issue would require more direct experimental verification.

Importantly, soil nutrients cannot solely explain the levels of *6Pgdh* found in this study. The minerals or nutrients can explain the variation across space, but they cannot explain the variation across seasons. It’s because even though the primary nutrients (nitrogen, potassium, phosphorus) can vary between seasons (Hu et al. 2022), there is little evidence of such being the case for the other minor nutrients and minerals. The shifts in *6Pgdh* levels might instead be a result of population demography and gene flow between local populations. Indeed, it has been suggested that bulbs are often colonized by small number of individuals or single gravid females, making founder effect an important determinant of genetic structuring. This could contribute to large differences and presumably erratic patterns of allele frequency changes in natural populations. Indeed, some of the population we have sampled were quite small, with high numbers of juvenile individuals, suggesting they had been founded recently. However, a recent field study found that colonization events are moderately common but, importantly, they do not seem to be associated with strong bottlenecks or founder effects (Przesmycka and Radwan 2023). A detailed study on the structure of genetic variation within and between bulb mite populations using genome-wide data would help to clarify this issue.

To conclude, the study shows that the *6Pgdh* polymorphism is indeed common in bulb mites. We also found significant influence of soil properties on the polymorphism levels. Additionally, we found that the patterns of *6Pgdh* polymorphism varied across locations and seasons, although there was no pattern to this change. Gene flow, driven by the migration of individuals between bulb mite populations, could be a crucial factor contributing to the observed variations in nature. In suspicion of a genotype-by-environment interaction for fitness, we looked at climatic variables such as temperature and precipitation, and their influence on the patterns of *6Pgdh*, but we found no evidence of such. Similarly, the life-history assays performed in the lab did not provide any evidence of temperature influencing fitness of the allelic variants.

In summary, soil properties can potentially explain the distribution of *6Pgdh* alleles of bulb mites in the wild, but not the spatio-temporal variation. Perhaps there are other environmental factors contributing to this variation or perhaps it’s the result of gene flow between populations. Complementing this study with additional experiments to test the effects of different factors on more traits (related to reproductive success) may help us understand more about how selection works in nature and about environment dependent balancing selection in general.

## Data Availability

The datasets generated during and/or analysed during the current study are available from the corresponding author on reasonable request.
